# Circulating exosomal immuno-oncological checkpoints and cytokines are potential biomarkers to monitor tumor response to anti-PD-1/PD-L1 therapy in non-small cell lung cancer patients

**DOI:** 10.3389/fimmu.2022.1097117

**Published:** 2023-01-18

**Authors:** Shayista Akbar, Afsheen Raza, Reyad Mohsin, Aladdin Kanbour, Shahnaz Qadri, Aijaz Parray, Abdul Rehman Zar Gul, Anite Philip, Suma Vijayakumar, Maysaloun Merhi, Shereena Hydrose, Varghese Philipose Inchakalody, Rajaa Al-Abdulla, Wafa Abualainin, Shaza Abu Sirriya, Issam Al-Bozom, Shahab Uddin, Omar Muhammad Khan, Mohamed Izham Mohamed Ibrahim, Ussama Al Homsi, Said Dermime

**Affiliations:** ^1^ College of Health and Life Sciences, Hamad Bin Khalifa University, Doha, Qatar; ^2^ Department of Medical Oncology, National Center for Cancer Care and Research, Hamad Medical Corporation, Doha, Qatar; ^3^ Translational Cancer Research Facility, Translational Research Institute, Academic Health System, Hamad Medical Corporation (HMC), Doha, Qatar; ^4^ Irma Lerma Rangel College of Pharmacy, Texas A&M University, Kingsville, TX, United States; ^5^ Neuroscience Institute, Academic Health System, Hamad Medical Corporation, Doha, Qatar; ^6^ Anatomical Pathology, Department of Laboratory Medicine and Pathology, Hamad Medical Corporation, Doha, Qatar; ^7^ Diagnostic Genomic Division, Solid Tumor Section, Department of Laboratory Medicine and Pathology, Hamad Medical Corporation, Doha, Qatar; ^8^ Diagnostic Genomic Division, Department of Laboratory Medicine and Pathology, Hamad Medical Corporation, Doha, Qatar; ^9^ Translational Research Institute and Dermatology Institute, Academic Health System, Hamad Medical Corporation, Doha, Qatar; ^10^ Laboratory Animal Research Center, Qatar University, Doha, Qatar; ^11^ Clinical Pharmacy and Practice Department, College of Pharmacy, QU Health, Qatar University, Doha, Qatar

**Keywords:** exosomes, NSCLC, biomarkers, immune-checkpoint inhibitors, immune-oncological-checkpoints, cytokines, follow-up

## Abstract

Immune checkpoint inhibitors (ICIs) including anti-PD-1 and anti-PD-L1 antibodies, have significantly changed the treatment outcomes of NSCLC patients with better overall survival. However, 15-40% of the patients still fail to respond to ICIs therapy. Identification of biomarkers associated with responses are mandated in order to increase the efficacy of such therapy. In this study we evaluated 27 serum-derived exosomal immuno-oncological proteins and 44 cytokines/chemokines before and after ICIs therapy in 17 NSCLC patients to identify surrogate biomarkers for treatment/monitoring patient stratification for maximum therapeutic benefit. We first confirmed the identity of the isolated exosomes to have their specific markers (CD63, CD81, HSP70 and CD91). We have demonstrated that baseline concentration of exosomal-PD-L1 (p<0.0001), exosomal-PD-L2 (p=0.0413) and exosomal-PD-1 (p=0.0131) from NSCLC patients were significantly higher than their soluble-free forms. Furthermore, the exosomal-PD-L1 was present in all the patients (100%), while only 71% of patients expressed tissue PD-L1. This indicates that exosomal-PD-L1 is a more reliable diagnostic biomarker. Interestingly, exosomal-PD-L2 expression was significantly higher (p=0.0193) in tissue PD-L1-negative patients compared to tissue PD-L1-positive patients. We have also shown that immuno-oncological proteins isolated from pre-ICIs treated patients were significantly higher in exosomes compared to their soluble-free counterparts (CD152, p=0.0008; CD80, p=0.0182; IDO, p=0.0443; Arginase, p<0.0001; Nectin-2, p<0.0001; NT5E, p<0.0001; Siglec-7, p<0.0001; Siglec-9, p=0.0335; CD28, p=0.0092; GITR, p<0.0001; MICA, p<0.0001). Finally, the changes in the expression levels of exosomal immuno-oncological proteins/cytokines and their correlation with tumor response to ICIs treatment were assessed. There was a significant downregulation of exosomal PD-L1 (p=0.0156), E-Cadherin (p=0.0312), ULBP1 (p=0.0156), ULBP3 (p=0.0391), MICA (p=0.0391), MICB (p=0.0469), Siglec7 (p=0.0078) and significant upregulation of exosomal PD-1 (p=0.0156) and IFN- γ (p=0.0156) in responding patients. Non-responding patients showed a significant increase in exosomal-PD-L1 (p=0.0078). Furthermore, responding-patients without liver-metastasis showed significant-upregulation of PD-1 (p=0.0070), and downregulation of ULBP1 (p=0.0137) and Siglec-7 (p=0.0037). Non-responding patients had significant-downregulation of ULBP3 (p=0.0317) in patient without brain-metastasis and significant-upregulation/downregulation of PD-L1 and ULBP3 (p=0.0262/0.0286) in patients with pulmonary-metastasis. We demonstrated for the first time that exosomal immuno-oncological proteins/cytokines are potential biomarkers to monitor response to ICIs therapy and can predict the clinical outcomes in NSCLC patients.

## Introduction

Lung cancer is the second most commonly diagnosed cancer and the main cause of cancer deaths, with an anticipated 2.2 million new cases and 1.8 million fatalities, accounting for around 11.4% of cancer diagnoses and over 18% of all cancer deaths worldwide (1). Non-small cell lung cancer (NSCLC) accounts for approximately 80-85% of total lung cancer cases (2, 3).

Immunotherapies, particularly immune checkpoint inhibitors (ICIs), that target programmed cell death protein 1 (PD-1)/programmed death-ligand 1 (PD-L1) signaling pathway, have prompted a paradigm shift in cancer treatment, demonstrating significant efficacy and long-term clinical benefits in different cancers, particularly melanoma and NSCLC (4, 5). The United States Food and Drug Administration (FDA) has approved immune checkpoint inhibitors for advanced NSCLC treatment, including anti-PD-1 (Pembrolizumab and Nivolumab) and anti-PD-L1 (Durvalumab, Atezolizumab) based on randomized trials that showed an overall survival benefit in this cohort of patients. Briefly, these are monoclonal antibodies that bind specifically to the PD-1 receptor on the T-cells, as a result of this, the T-cell response is augmented or increased along the targeted tumor cells and hence, these antibodies prevent tumor cells from escaping the immune system by inhibiting the interaction between PD-1 and PD-L1 (6). Despite the fact that ICIs have shown better overall survival in NSCLC (4, 5, 7), the response rate is approximately 20-30% (8). Therefore, the need to identify biomarkers that can help to monitor treatment response and selection of patients for maximum therapeutic benefits is critical.

Several clinical studies in NSCLC have associated treatment response in patients under ICIs with predictive biomarkers on tissues, such as PD-L1 expression on tumor tissues (9). However, tissue-based biopsies have several drawbacks, including the requirement of an invasive procedure, the inability to monitor changes in the tumor molecular profile during therapy and remote localization and heterogeneity of PD-L1 expression within the tumor (10). Recent studies have reported the role of exosomes as promising biomarkers for several cancers (11–14). Exosomes are nano-sized vesicles with a diameter of 30-150 nm, secreted by most cell types through a well-defined endosomal route (15, 16). The exosomes are protected by a lipid bilayer membrane and carry various bioactive molecules, including nucleic acids, lipids and proteins. Further, it is now well established that cancer cells actively release exosomes with cancer-promoting content to mediate intercellular communication within the tumor microenvironment (TME) and thus play a key role in tumor progression (17). Studies suggest that exosomes possess high biomarker potential, mainly due to their ability to embed and represent biomolecule cargo from the originating cells (18–20) and their reproducible detectability in most body fluids (21). Another unique feature of exosomes is their stability in circulation, as their biomolecule cargo is protected from enzymatic degradation in biofluids with a cholesterol-rich lipid bilayer membrane (22, 23). Additionally, when compared to whole blood, exosomal markers possess higher sensitivity and specificity (24, 25).

The immuno-oncological proteins are important regulators of T-cells and Natural Killer (NK) cells that cause either inhibition or stimulation of such cells, thus affecting the antitumor immune responses. Many studies have reported that tumor-derived exosomes (TEXs) are enriched in immuno-oncological proteins, including T-cell immune checkpoints such as PD-L1, cytotoxic T-lymphocyte-associated antigen 4 (CTLA-4), T-cell immunoglobulin mucin-3(TIM-3) and NK immune checkpoints such as MHC class I chain-related protein A (MICA), MHC class I chain-related protein B (MICB), UL16 binding protein 1 (ULBP1) and UL16 binding protein 2 (ULBP2) (26–29). Additionally, pro- and anti-inflammatory cytokines/chemokines involved in tumor cell migration and progression such as interleukin 2 (IL-2), interleukin 4 (IL-4), interleukin 10 (IL-10), interleukin 18 (IL-18), interleukin 33 (IL-33), tumor necrosis factor alpha (TNF-α), transforming growth factor-beta (TGF-β) and macrophage colony-stimulating factor (M-CSF) have been reported to be preferentially enriched within exosomes (30–32). Therefore, exosomes can serve as important biomolecules for disease monitoring and treatment dynamics. The role of various exosomal biomarkers in disease dynamics has been documented for various cancers (33). However, a limited number of studies on the expression of exoPD-1, exoPD-L1 and few cytokines in exosomes of NSCLC patients under ICIs treatment have been reported. To our knowledge, no study on extensive profiling of exosomal immuno-oncological proteins and cytokines in NSCLC patients pre- and post-ICIs treatment has been conducted. Therefore, the main aim of this study is to identify the role of T-cell immune checkpoint molecules, NK immune checkpoint markers and cytokines derived from exosomes of ICIs treated NSCLC patients as biomarkers of response to anti-PD-1/PD-L1 therapy.

## Materials and methods

### Patients and sample collection

The prospective study was conducted at the National Center for Cancer Care and Research (NCCCR), Hamad Medical Corporation (HMC), Doha, Qatar from September 2020-June 2022. A total of 17 locally advanced or metastatic NSCLC patients eligible for anti-PD-1/PD-L1 monotherapy or combined chemo-immunotherapy, as their standard treatment protocol were enrolled. Written informed consent was signed by all the subjects who donated blood as per the Declaration of Helsinki principles.

10 ml of blood were collected in serum separator vacutainer tubes from eligible participants at two-time points: (a) before initiation of immunotherapy and (b) at the time of PET CT imaging (4-6 months after immunotherapy-routinely done for determination of clinical response). The collected blood was allowed to clot at room temperature for approximately 15-30 minutes and then centrifuged at 1300 x g for 10 minutes to collect serum. The serum was aliquoted and stored at -80°C until further analysis.

### Measurement of PD-L1 expression in tumor tissue

Tissue PD-L1 expression was performed in the CAP accredited Department of Laboratory Medicine and Pathology (DLMP), HMC, Qatar as part of routine diagnostic testing. PD-L1 expression was assessed on formalin-fixed, paraffin-embedded (FFPE) tissue from NSCLC patients. The PD-L1 expression was assessed, according to the manufacturers’ instructions, by qualitative immunohistochemical assay (DAKO PD-L1 IHC 22C3 pharmDx) using monoclonal mouse anti-PD-L1, Clone 22C3 on Automated Autostainer Link 48 (Dako, USA). Briefly, following incubation with the primary monoclonal antibody to PD-L1 or the Negative Control Reagent (NCR), specimens were incubated with a Linker antibody specific to the host species of the primary antibody, and then incubated with a ready-to-use visualization reagent consisting of secondary antibody molecules and horseradish peroxidase molecules coupled to a dextran polymer backbone. The enzymatic conversion of the subsequently added chromogen results in the precipitation of a visible reaction product at the site of the antigen. The color of the chromogenic reaction was modified by a chromogen enhancement reagent. The specimen was then counterstained, and cover slipped. The entire slide was evaluated by pathologist using a light microscope objective of 10-40X. To ensure run quality control, the slides were examined in the order H&E, control cell line slide, positive control tissue slides, negative control tissue, patient tissue slide stained using the Negative control reagent (NCR) and patient tissue slide stained using the PD-L1 primary antibody slides to determine the validity of the staining run and enable assessment of the staining of the sample tissue. For PD-L1 scoring, a minimum of 100 viable tumor cells, negative and positive controls are required for quality control and test validity. PD-L1 protein expression was determined by using Tumor Proportion Score (TPS), which is the percentage of viable tumor cells showing partial or complete membrane staining. The specimen is considered to have PD-L1 expression (weak positivity) if TPS ≥ 1% of the viable tumor cells exhibit membrane staining at any intensity, high PD-L1 expression (strongly positive) if TPS ≥ 50% of the viable tumor cells exhibit membrane staining at any intensity. The intensity was evaluated as follows: No staining scored as “0”, Weak staining as “1+”, Moderate staining as “2+”, and Strong staining as “3+”. The specimen was considered PD-L1+ if ≥1% of the viable tumor cells exhibit membrane staining at any intensity (regardless of degree intensity, 1+, 2+, 3+), PD-L1 strong positive if ≥50% staining, PD-L1 weak positive if ≥1% but <50% (DAKO PD-L1 IHC 22C3 pharmDx datasheet).

### Exosome isolation and purification

Exosomes were isolated from 500 µl of serum samples by differential centrifugation and ultracentrifugation as described by Théry et al. (34) with some modifications. Differential ultracentrifugation method is currently the gold standard of exosome enrichment (35) and several studies have reported the use of this technique (13, 36). Briefly, the viscosity of the serum was reduced by diluting it with PBS (1:2) and centrifuging at 400 x g for 10 minutes at 4°C. To remove cell debris, the extracted supernatant was first centrifuged at 5000 x g for 20 minutes followed by second centrifugation at 20,000 x g for 30 minutes to pellet microparticles. The clarified supernatant was transferred to 1.5 ml Ultra Microtubes (Thermo Scientific) and ultracentrifuged using Fixed Angle Microtube Rotor at 100,000 x g for 4 hours to pellet exosomes (Hitachi, CP100NX). The collected exosome pellets were resuspended in PBS and subjected to ultracentrifugation at 100,000 x g for another 4 hours to isolate purified exosomes. Final exosome pellets were re-suspended in 1 X PBS and stored at -20°C until further experiments.

### Western blot analysis

For total protein extraction from exosomes, lysis was performed with RIPA buffer (Thermo Scientific) containing protease inhibitor cocktail and Laemmli buffer (Bio-Rad). The total protein concentration was quantified using a BCA protein assay kit (Thermo Scientific) following the manufacturer’s instructions. Briefly, approximately, 30 μg of protein extracts were electrophoresed on a 4-15% Mini protean pre-cast polyacrylamide gel (Bio-Rad) and transferred to PVDF membrane (Bio-Rad). The membrane was blocked with 5% nonfat dry milk (Cell Signaling) for 1 hour and immunoblotted overnight at 4 °C with primary antibodies to CD63 (ExoAB-CD63A-1 SBI system Biosciences), CD81 (ab59477, Abcam), Hsp70 (EXOAB-Hsp70A-1), CD91 (ab20384, Abcam) and GAPDH (ab8245, Abcam). After incubation, anti-rabbit (7074S, Cell Signaling Technology/EXOAB-HRP) or anti-mouse (ab97040, Abcam) secondary antibodies were applied for 1 hour. The membrane was then visualized by Clarity western ECL substrate (Bio-Rad). Images were acquired using the Bio-Rad Chemidoc MP Imaging system.

### Transmission electron microscopy

The morphology and size of exosomes were determined by transmission electron microscopy (TEM). A 5 μl exosome suspension was carefully placed on Formvar/carbon 200 mesh copper grid (Ted Pella, Inc.) for 20 minutes, followed by fixation with 4 Glutaraldehyde and 2% Formaldehyde fixative mixture for 5 minutes. After three times washing with PBS, the grids were negatively stained with 2% uranyl acetate for 5 minutes and washed again three times. The samples were dehydrated with ethanol gradient solutions and then placed in a vacuum desiccator for drying. The morphology of exosomes was visualized under a JEM-1200EX TEM-SCAN electron microscope (JEOL, Akishima, Japan) and the images were analyzed by ImageJ software.

### Dynamic light scattering analysis

The hydrodynamic size of exosomes was measured using Dynamic light scattering (DLS) Zetasizer (Malvern Panalytical Limited, UK). Briefly, the exosomes were diluted with 1 X PBS, transferred into a sub-micro Quartz cuvette, and placed in a DLS device. Laser and temperature equilibrium of the device was monitored with measurements performed at a constant temperature of 24°C. The radius or percent intensity was used to measure the size or homogeneity of exosomes, respectively.

### Measurement of T cells and NK derived immune checkpoints and cytokines for exosomal biomarker concentrations

A total of 72 biomarkers of T cells and NK derived immune checkpoints and cytokines including BTLA, HVEM, CD152 (CTLA4), CD28, CD80, GITR, IDO, CD27, LAG-3, PD-L1, PD-L2, PD-1, CD137(4-1BB), TIM-3, Arginase-1, E-Cadherin, MICA, MICB, Nectin 2 (CD112), NT5E (CD73), PVR (CD155), Siglec-7, Siglec-9, Tactile (CD-96), ULBP1, ULBP3, ULBP4 and FGF basic, G-CSF, GM CSF, GRO-α, HGF, IFN-α2, IFN-γ, IL-1α, IL-1β, IL-1ra, IL-2Rα, IL-2, IL-3, IL-4, IL-5, IL-6, IL-7, IL-8, IL-9, IL-10, IL-12 (p70), IL-12 (p40), IL-16, IL-17, IL-18, IP-10, LIF, MCP-3, MCP-1, MCP-3, M-CSF, MIF, MIG, MIP-1α, MIP-1β, β-NGF, PDGF-BB, RANTES, SCF, SCGF-β, SDF-1α, TNF-α, TNF-β, TRAIL, VEGF were tested from purified exosomes using the following kits; Procarta Plex Human Immuno-Oncology Checkpoint Marker Panel 1 (Thermo scientific); Procarta Plex Human Immuno-Oncology Checkpoint Marker Panel 2 (Thermo scientific) and Bio-Plex Pro Human Cytokine Screening 48-plex panel (Bio-Rad).

The assay protocol involved the dilution of exosomes in Universal Assay buffer (1:20) and added to a 96-well plate loaded with 50 μl of antibody-coupled beads. After overnight incubation, the beads were washed to remove unbound proteins and incubated with 25 μl of 1 X detection antibodies for 30 minutes at room temperature on a shaker. The beads were washed to remove unbound detection antibodies, followed by the addition of Streptavidin-PE with subsequent washing off the excess streptavidin-PE. The beads were resuspended in reading buffer and fluorescent signals were detected in Luminex Bio-Plex 200 system (Bio-Rad). Acquisition and data analysis were performed by Bio-plex Manager TM version 6.2 software. A standard curve was produced using a premixed antigen standard and concentrations were calculated based on seven-point standard curves using a five-parametric fit algorithm in xPONENT v4.0.3.

### Statistical analysis

Statistical analysis was performed using GraphPad Prism 7 software. The data were expressed as mean ± SD or median with interquartile range. The differential expression level of markers between exosomes and serum levels and pre- and post-treatment were analyzed using Wilcoxon Signed Rank test. Mann-Whitney U test was used to compare expression level of exosomal markers with tissue-PD-L1 status and between responders and non-responders. ROC curve analysis was used to calculate the cut-off values for exosomal markers. Western blot and transmission electron microscopy data was processed by using ImageJ software. All statistical analysis was two-tailed with p<0.05 considered significant.

## Results

### Patient characteristics

In this pre-post prospective clinical study, 17 locally advanced or metastatic NSCLC patients eligible for immunotherapy as a standard treatment protocol were included. Out of these, 82% of patients were male while 18% were female. The mean age of patients was 60 years. 76% of the patients were diagnosed with adenocarcinoma subtype, while the remaining 24% were diagnosed with squamous cell carcinoma. Twelve of the patients expressed tissue-PD-L1 with tumor proportion score of ≥1% and were designated as tissue-PD-L1+ group while five were tissue-PD-L1-. In terms of treatment, monotherapy with anti-PD-1 was given with Pembrolizumab for 35% while Nivolumab was given to 6% of the patients respectively. In addition to this, 12% of the patients were given anti-PD-L1(Durvalumab) while 47% of the patients received a combination of chemo-immunotherapy with Pembrolizumab/Atezolizumab plus Carboplatin, Pemetrexed. All these patients were clinically evaluated 4-6 months after the treatment *via* imaging with PET CT to assess treatment response. Patient demographics and clinical characteristics are detailed in **Table 1**.

**Table 1:** Baseline demographics and clinical characteristics of patients.

Patient Characteristics	Number of patients (n=17)	%
Age		
Median (year, range)	60	
<60	9	53
≥60	8	47
Gender		
Male	14	82
Female	3	18
Smoking history		
Never	5	29
Current to former	12	71
Histology		
Adenocarcinoma	13	76
Squamous cell carcinoma	4	24
ECOG PS		
0-1	14	82
≥2	3	18
Genetic alterations		
EGFR-ALK Wild type	14	82
EGFR Mutated	0	0
ALK Mutated	1	6
EGFR-ALK Inconclusive	2	12
Other alterations(KRAS, BRAF, AKT, FGFR3, MET, PIK3CA, ERBB3)	6	35
PD-L1 TPS		
<1%	5	29
1% to 49%	4	24
>50%	8	47
Brain metastasis		
Present	7	41
Absent	10	59
Liver Metastasis		
Present	3	18
Absent	14	82
Pulmonary Metastasis		
Present	10	59
Absent	7	41
Treatment type		
Pembrolizumab	6	35
Nivolumab	1	6
Durvalumab	2	12
Pembrolizumab +Carboplatin +Pemetrexed	8	47

### Characterization of serum-derived exosomes from NSCLC patients

**Figure 1:**
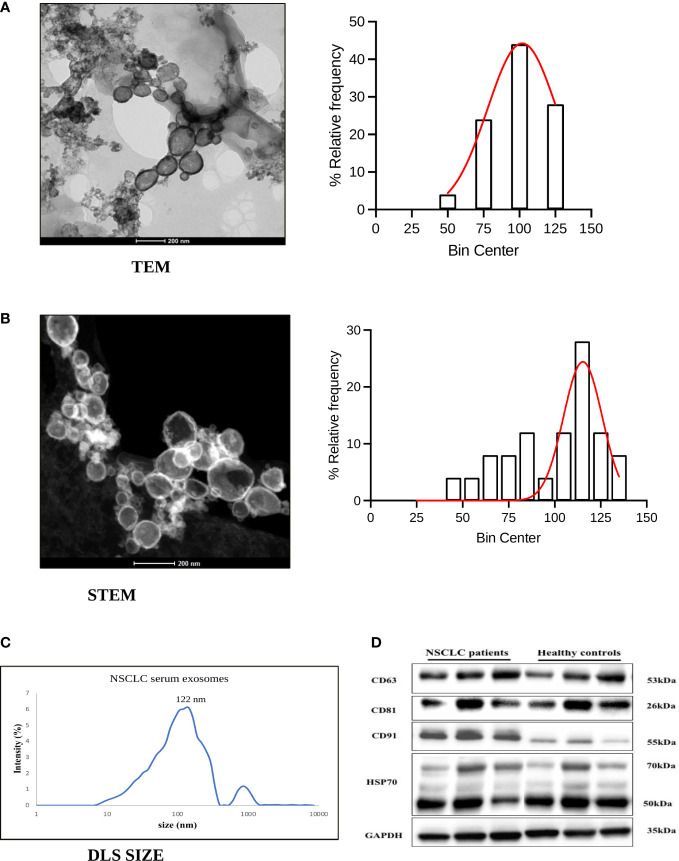
Characterization of exosomes derived from serum of NSCLC patients. **(A, B)** Representative figure and histogram with the gaussian curve (bin centre=25nm) of transmission electron microscopy (TEM) and Scanning Transmission electron microscopy (STEM) showing characterization for exosome morphology and size distribution of exosomes derived from NSCLC patients **(C)** Hydrodynamic size measurement of exosomes by Dynamic Light Scattering (mean value 122nm). **(D)** Representative western blot images showing expression of exosome-specific markers CD63, CD81, HSP70, and lung cancer protein CD91 among NSCLC patients and healthy controls.

To verify the exosomes isolated from the serum of NSCLC patients and healthy volunteers, we validated them in terms of morphology, size, and specific markers. The biophysical characterization by TEM and STEM showed their morphology as spherical, membrane-bound vesicles with an average size of 100 nm and 98.3 nm respectively (**Figures 1A, B**) which is an expected size range. The hydrodynamic size measurement by dynamic light scattering validated the average size of the maximum exosome population at 122 nm (**Figure 1C**). Further, these exosomes were verified in terms of exosome specific markers CD63, CD81 and HSP70 by western blotting (**Figure 1D**). In addition, CD91, reported in our recently published study (37) as a specific marker expressed on NSCLC derived exosomes, was also detected thereby indicating that the extracted exosomes were derived from NSCLC cells (**Figure 1D**). Altogether, these results show that our exosomes were successfully generated from the serum samples of NSCLC patients.

### Expression of PD-L1, PD-L2 and PD-1 from serum-derived exosomes compared to soluble free forms and tumor biopsies of NSCLC patients

**Figure 2:**
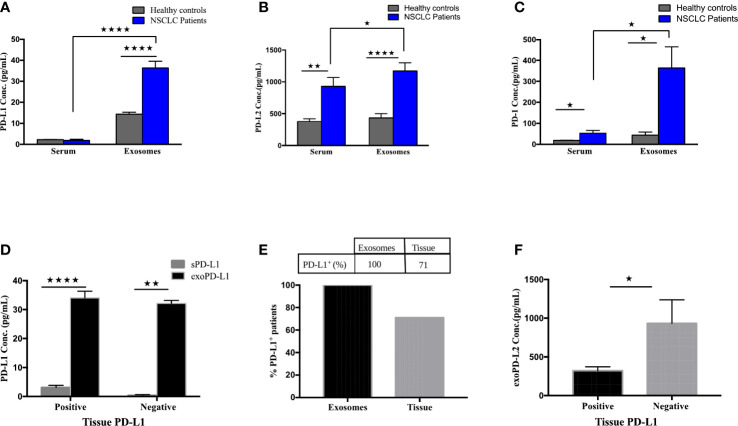
Expression of PD-L1, PD-L2 and PD-1 in circulating exosomes compared to free soluble forms or tumor biopsies. **(A)** PD-L1 levels in serum-derived exosomes of NSCLC patients compared with free soluble forms in the serum (p<0.0001). **(B)** PD-L2 levels in serum-derived exosomes of NSCLC patients compared with free soluble PD-L2 in the serum (p=0.0413). **(C)** PD-1 levels in serum-derived exosomes of NSCLC patients compared with free soluble PD-1 in the serum (p=0.0131). **(D)** Expression of exoPD-L1 and serum PD-L1 between tissue-PD-L1 positive and tissue-PD-L1 negative NSCLC patients (for positive grp, p<0.0001; for negative patients, p=0.0079). **(E)** Percentage of PD-L1 positive patients compared between circulating exosomes and tumor samples. **(F)** exoPD-L2 expression between tissue-PD-L1 positive and negative patients (p=0.0193). * significant; ** moderate significant; **** Highly significant.

To determine the expression of exoPD-L1, PD-L2 and PD-1 and their soluble free forms from the serum of NSCLC patients, their baseline levels were quantified by multiplexed bead immunoassay. The exoPD-L1 concentration of NSCLC patients was significantly higher (p<0.0001) than that of healthy controls, while the sPD-L1 profile of NSCLC patients and healthy controls did not show any significant variations (**Figure 2A**). We also compared the concentration of exoPD-L1 with sPD-L1 in the serum of NSCLC patients and found that the exoPD-L1 levels (mean=45.25 pg/ml, n=17) were dramatically higher than sPD-L1(mean=2.32 pg/ml, n=17) (**Figure 2A**). Similarly, exoPD-L2 and exoPD-1 levels from NSCLC patients were significantly higher (p=0.0413; p=0.0131) than that of their respective soluble forms as well as healthy controls (p<0.0001; p=0.0258) (**Figures 2B, C**). Moreover, by calculating an optimal cut-off based on ROC curve analysis, we found that exoPD-L1 cut-off value >20.36 pg/ml has a sensitivity and specificity of 100%, exoPD-L2 cut-off value >686.2pg/ml has a sensitivity of 93% and specificity 90% while exoPD-1 cut-off value >50.64 pg/ml has a sensitivity 68.75% and specificity 70%.

Furthermore, the expression of exoPD-L1 and sPD-L1 was compared between tissue-PD-L1 positive (n=12) and negative patient groups (n=5) and showed that exoPD-L1 was significantly higher than sPD-L1 in both tissue-PD-L1 positive (p<0.0001) and negative (p=0.0079) groups (**Figure 2D**). Further, when compared with tissue-PD-L1, our results show that exoPD-L1 was present in all the patients (100%) while only 71% of patients were tissue-PD-L1+ (**Figure 2E**). With regards to the comparison of tissue-PD-L1 positive and negative groups with exoPD-L2 expression, interesting results were observed with exoPD-L2 levels significantly higher in tissue-PD-L1- patients (p=0.0193) as compared to positive patients (**Figure 2F**) indicating that compared to tissue sample, exoPD-L1 and exoPD-L2 are better biomarkers for detection of PD-L1/PD-L2. Thus, we conclude that detecting and quantifying exosomal PD-L1, PD-L2 and PD-1 are considerably easier than soluble forms in the serum as well from biopsies.

### Differential expression of exosomal PD-L1/PD-1 according to treatment response

**Figure 3:**
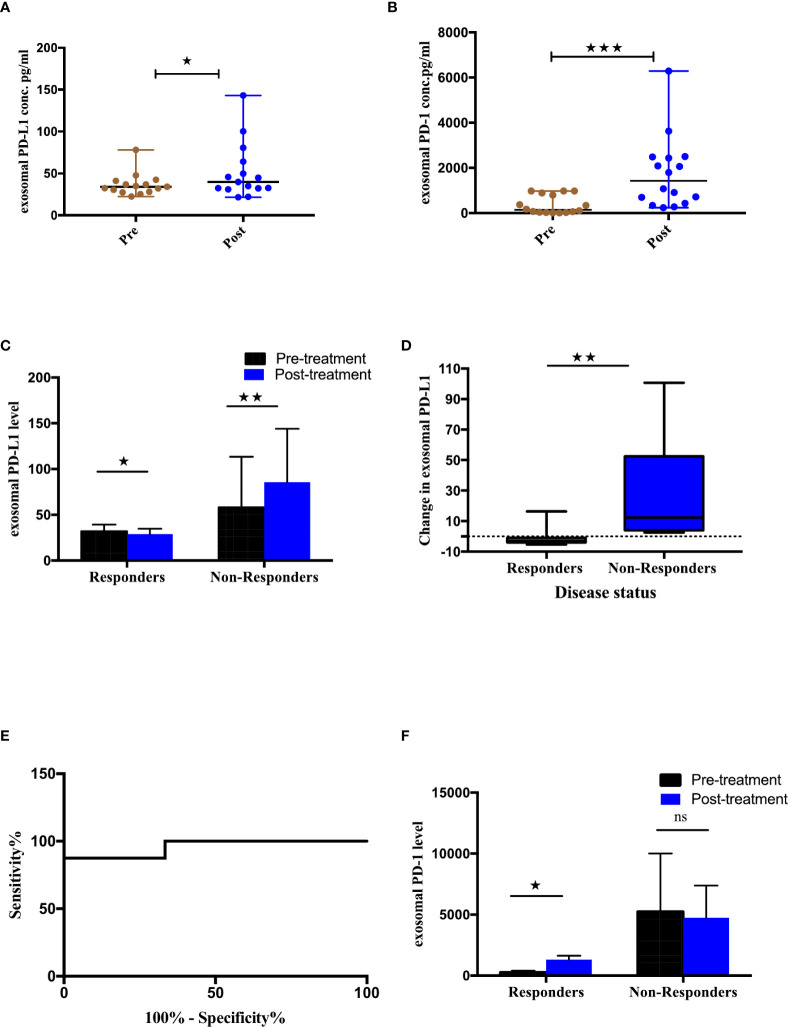
Association of exosomal PD-L1/PD-1 levels and clinical outcome. **(A, B)** Expression of exosomal PD-L1 and PD-1 levels pre and post ICI therapy respectively. **(C)** Comparison of exoPD-L1 expression in NSCLC patients before and after treatment grouped according to disease status. Two-tailed Wilcoxon matched-pairs signed rank test (*p=0.0156, **p=0.0078) **(D)** Fold change of exoPD-L1 in responders and non-responders 4-6 months after treatment commencement, to baseline. Mann Whitney(**p=0.0026) **(E)** Changes in exoPD-L1 levels analyzed using a ROC curve in responders relative to non-responders (AUC = 0.958, CI 95% = 0.865-1.051, SE = 0.047, p = 0.0015). **(F)** Comparison of exoPD-1 expression in NSCLC patients before and after treatment, grouped according to disease status. Two-tailed Wilcoxon matched-pairs signed rank test (*p=0.0156). **** Highly significant; ns non-significant.

To identify the association between differential expression of exosomal PD-L1 or PD-1 and tumor response, the variation in the expression of exoPD-L1/PD-1 extracted from the serum of NSCLC patients before and after immunotherapy was assessed. A significant difference in the exosomal PD-L1 (p=0.0294) as well PD-1 (p=0.0006) levels before and after the treatment was observed (**Figures 3A, B**). Further, the patients were stratified as responders and non-responders based on the clinical data of tumor response detected on PET-CT 4-6 months after the initiation of treatment. As shown in **Figure 3C**, exoPD-L1 levels were found to decrease significantly in patients exhibiting complete or partial response (n=8) (p=0.0156) while for patients experiencing progression (non-responding) (n=9), exoPD-L1 levels were found to be significantly increased (p=0.0078). Moreover, the fold change in the expression level of exoPD-L1 was also found to be significantly increased in progressing (non-responding) patients (p=0.0026) (**Figure 3D**). ROC curve analysis of changes in levels of exoPD-L1 in responders compared with non-responders, demonstrated 87% sensitivity and 100% specificity thus showed good discrimination between responding and non-responding patients (p=0.0015) (**Figure 3E**). Interestingly, significant upregulation of exoPD-1 was observed in responding patients after ICIs therapy (p<0.0156) (**Figure 3F**). Our results showed that downregulation of exoPD-L1 levels and upregulation of exoPD-1 levels were associated with tumor response, whereas upregulation of exoPD-L1 was associated with tumor progression.

### Immune-oncology markers expression in serum versus circulating exosomes of NSCLC patients

**Figure 4:**
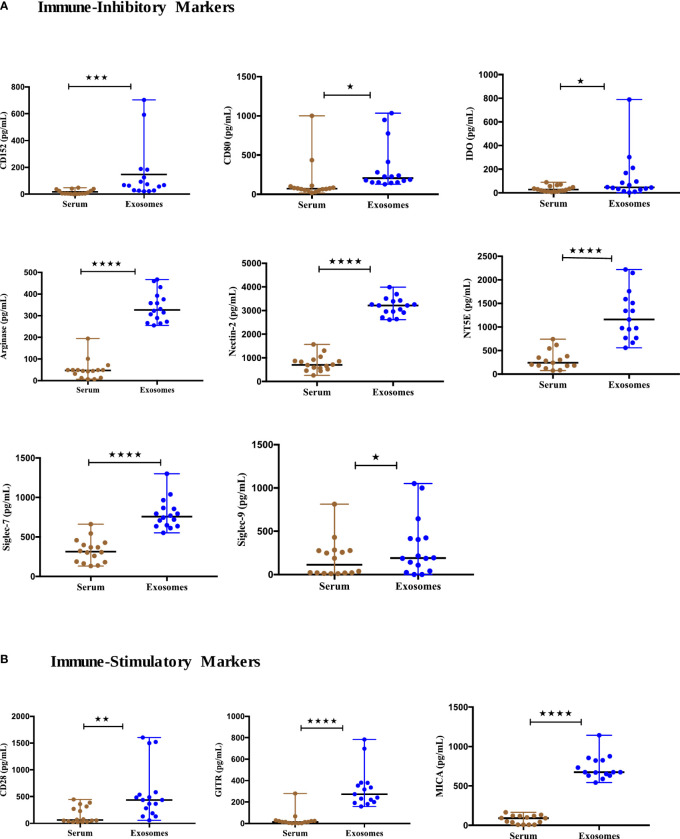
Expression of immune-oncology biomarkers from circulating exosomes compared to their soluble forms in serum of NSCLC patients. **(A)** Significantly higher Immune inhibitory protein levels **(B)** Significantly higher Immune-stimulatory protein levels in serum-derived exosomes of NSCLC patients (n=17) compared to their levels in serum. Two-tailed Wilcoxon matched-pairs signed rank test (*p<0.05, **p<0.01, ***p<0.001, ****p<0.0001).

To assess the utility of other immuno-oncological proteins studied from exosomes as tumor biomarkers, we profiled the baseline expression of 27 exosomal and soluble immuno- oncological markers from the serum of 17 NSCLC patients (Supplement 1). Our results showed the levels of immune inhibitory checkpoints including CTLA4 (CD152) (p=0.0008), CD80 (p=0.0182), IDO (p=0.0443), Arginase-1 (p<0.0001), Nectin-2 (CD112) (p<0.0001), NT5E (CD73) (p<0.0001), Siglec-7 (p<0.0001) and Siglec-9 (p=0.0335) were significantly higher in exosomes (**Figure 4A**). Furthermore, significant upregulation of immune stimulatory proteins, including CD28 (p=0.0092), GITR (p<0.0001), and MICA (p<0.0001) was observed in exosomes as compared to their levels in serum (**Figure 4B**). The remaining tested markers didn’t show significant differences between exosomal and serum levels and some proteins were below the detection range and were thus omitted from the analysis. Therefore, the higher expression of these T cells and NK immune checkpoint biomarkers in exosomes indicates the significance of exosomes as better samples for tumor profiling as compared to serum.

### Differential expression of T cells immune checkpoint from exosomes of NSCLC patients pre- and post-ICIs therapy

**Figure 5:**
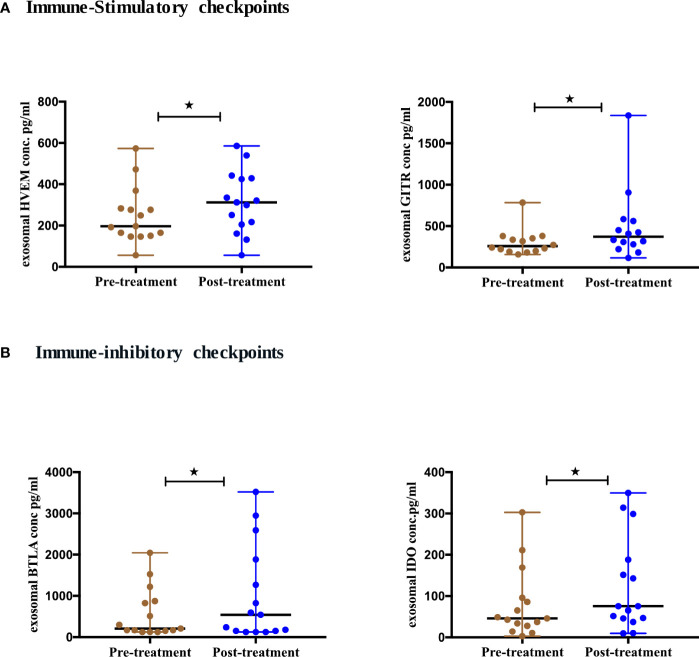
Differential expression of exosomal immune- checkpoints markers post-ICI therapy. **(A)** Immune-stimulatory checkpoints **(B)** Immune-inhibitory checkpoints from serum-derived exosomes of NSCLC patients (n=17) before and after anti-PD-1/PD-L1 checkpoint blockade immunotherapy. Two-tailed Wilcoxon matched-pairs signed rank test (*p<0.05).

Immune-checkpoint markers play a key role in T cell regulation leading to either T cell exhaustion or stimulation. We, therefore, evaluated the expression of circulating exosomal immune-checkpoint markers, with treatment dynamics in NSCLC patients (n=17). We compared the levels of these biomarkers pre- and post-ICIs therapy (Supplement 2). 4/14 checkpoint markers showed statistical significance (**Figure 5**). The expression of 2 stimulatory immune checkpoints including HVEM (p=0.0353) and GITR (p=0.0295) were significantly upregulated after ICIs therapy (**Figure 5A**). Additionally, 2 of the inhibitory immune checkpoints including BTLA (p=0.0353) and IDO (p=0.0413) were also significantly upregulated after ICIs therapy (**Figure 5B**).

### Differential expression of NK immune checkpoint markers from exosomes of NSCLC patients pre- and post-ICIs therapy

**Figure 6:**
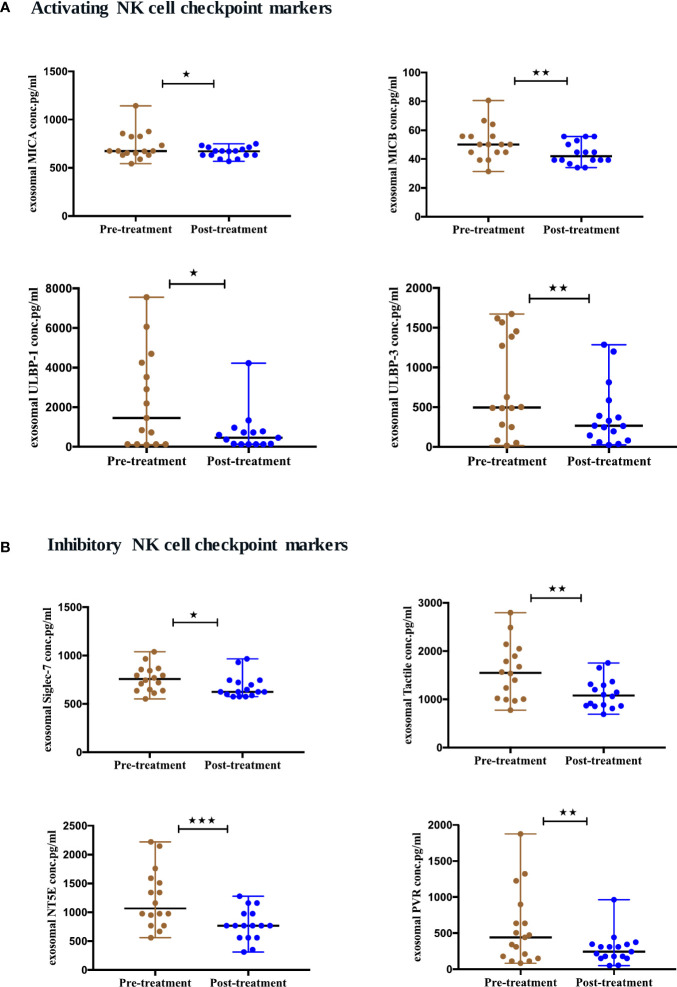
Differential expression of exosomal NK cell checkpoint markers post-ICI therapy. **(A)** Activating NK cell checkpoint markers **(B)** Inhibitory NK cell checkpoint markers from serum-derived exosomes of NSCLC patients (n=17) before and after anti-PD-1/PD-L1 therapy. Two-tailed Wilcoxon matched-pairs signed rank test (*p<0.05, **p<0.01, ***p<0.001).

To evaluate the expression of natural killer (NK) cells checkpoint markers from serum-derived exosomes of NSCLC patients pre- and post-ICIs therapy, a panel of 14 NK markers was quantified at baseline and 4-6 months after the initiation of ICIs therapy. We showed that 8 NK cell markers were differentially expressed after ICIs treatment. The activating NK cell checkpoint markers including MICA (p=0.0457), MICB (p=0.0029), ULBP1 (p=0.0215) and ULBP3 (p=0.0063) were downregulated after ICI therapy (**Figure 6A**). Moreover, the inhibitory NK cell markers including siglec-7 (p=0.0250), tactile (CD96) (p=0.0027), NT5E (CD73) (p=0.0004) and PVR (p=0.0042) were also downregulated post-ICIs therapy (**Figure 6B**).

### Correlation of differential expression of exosomal immune-oncology markers and treatment outcome

**Figure 7:**
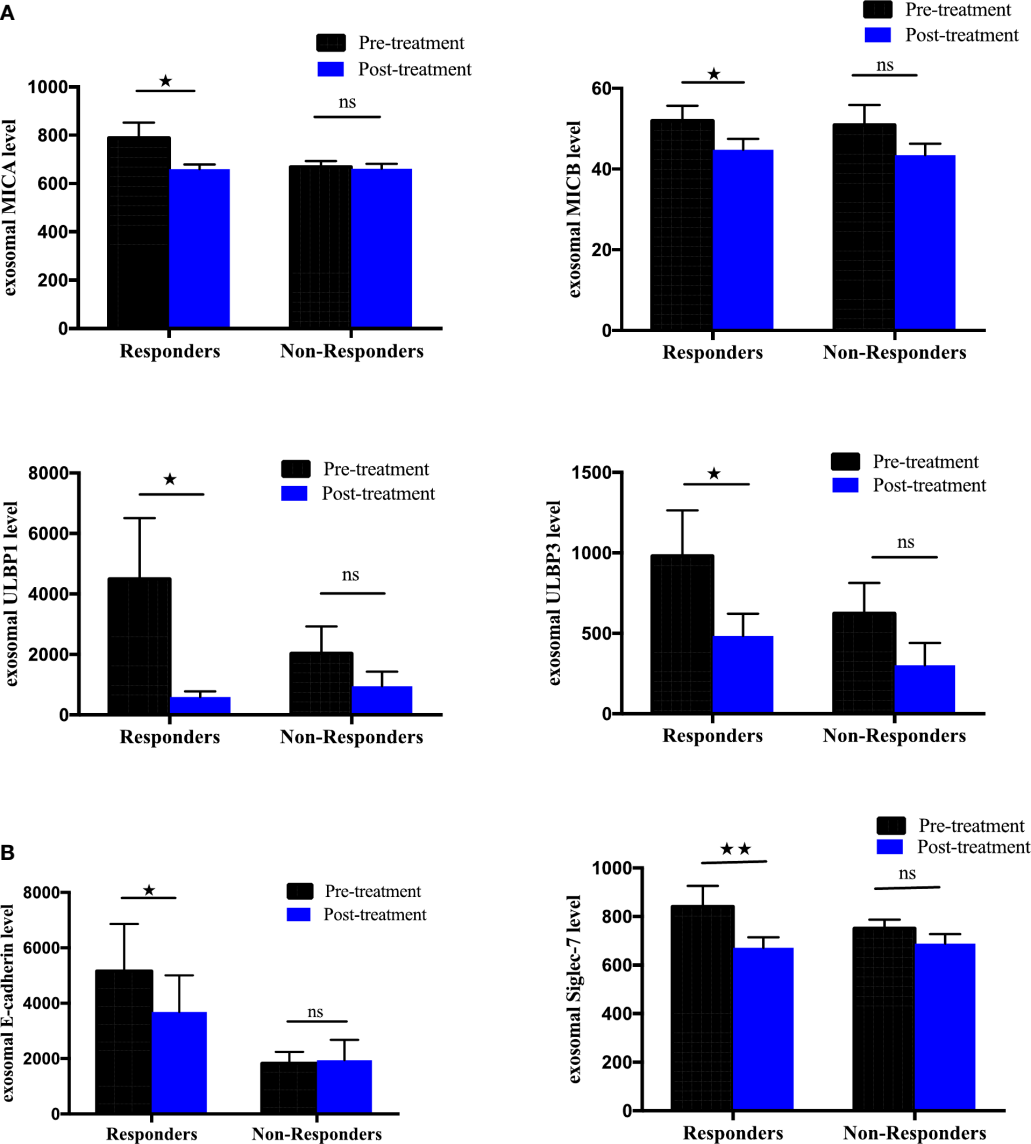
Differential expression of exosomal immune-oncology markers stratified NSCLC patients according to clinical outcome. Association of change in **(A)** exosomal immune-stimulatory markers including MICA (p=0.0391), MICB (p=0.0469), ULBP1 (p=0.0156), ULBP3 (p=0.0391) and **(B)** exosomal immune-inhibitory markers including E-Cadherin (p=0.0312), Siglec-7 (p=0.0078) evaluated before and after ICI therapy in NSCLC patients grouped according to disease status (responders, n= 8; non-responders=9. Wilcoxon matched-pairs signed-rank test. P-values <0.05 were considered statistically significant. * significant; ** moderate significant; ns non-significant.

**Figure 8:**
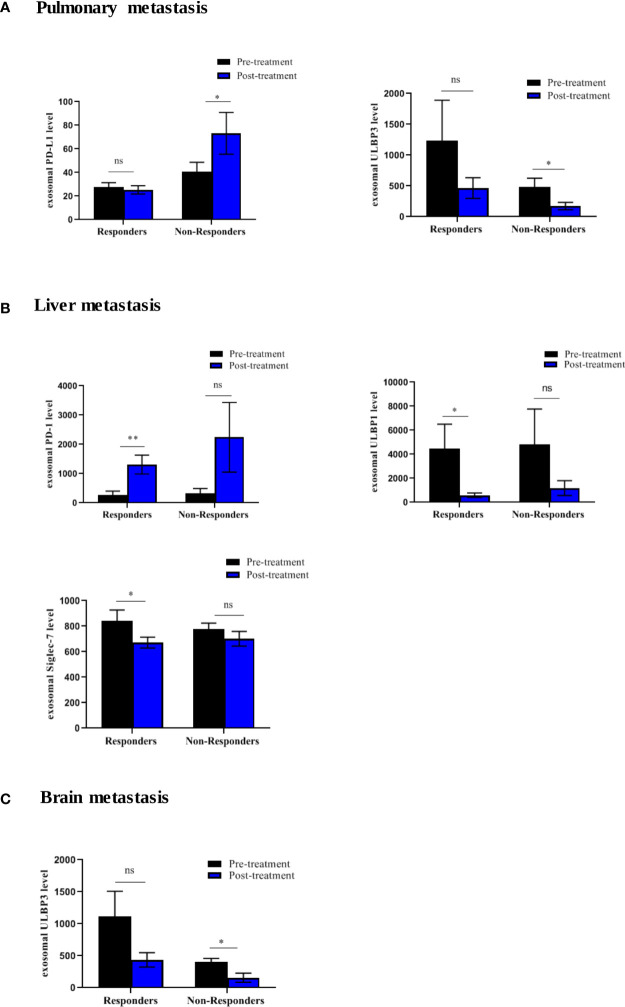
Differential expression of exosomal immune-oncology markers stratified according to metastasis in NSCLC patients. **(A)** Significant upregulation of PD-L1 (p=0.0262) and downregulation of ULBP3 (p=0.0286) in non-responding patients with pulmonary metastasis. **(B)** Significant upregulation of PD-1 (p=0.0070) and downregulation of ULBP-1(p=0.0137), Siglec-7 (p=0.0371) in responding patient without liver metastasis. **(C)** Significant downregulation of ULBP3 (p=0.0317) in non-responding patients without brain metastasis. * significant; ** moderate significant; ns non-significant.

To further elucidate the association between changes in the expression of exosomal immuno-oncology markers and tumor response, the patients were stratified into responding and non-responding groups based on the clinical data of tumor response detected on PET-CT 4-6 months after the initiation of treatment. Our results showed significant downregulation of 4 immune-stimulatory markers including MICA (p=0.0391), MICB (p=0.0469), ULBP1 (p=0.0156) and ULBP3 (p=0.0391) in responders after ICIs therapy (**Figure 7A**). On the other hand, 2 immuno-inhibitory markers including E-cadherin (p=0.0312) and Siglec-7 (p=0.0078) were significantly downregulated in responders as shown in (**Figure 7B**). However, no significant difference was found in non-responding patients (**Figures 7A, B**). This indicates that the tumor response was correlated with a significant change in some circulating exosomal immuno-oncological protein levels. Furthermore, comparison of markers with metastasis, we showed a significant upregulation of PD-L1 (p=0.0262) and downregulation of ULBP3 (p=0.0286) in non-responding patients with pulmonary metastasis (**Figure 8A**). In patients without brain metastasis, ULBP3 (p=0.0317) was found to be significantly downregulated post-treatment in non-responding patients (**Figure 8C**). Moreover, in patients without liver metastasis, significant upregulation of PD-1 (p=0.0070), and downregulation of ULBP1 (p=0.0137) and Siglec-7 (p=0.0371) were observed post-treatment in responding patients (**Figure 8B**). Correlation of altered genes, smokers vs. non-smokers in responding and non-responding patients did not show any significant difference (data not shown).

### Differential expression of cytokines and chemokines from serum-derived exosomes pre- and post-ICIs therapy and disease outcome

**Figure 9:**
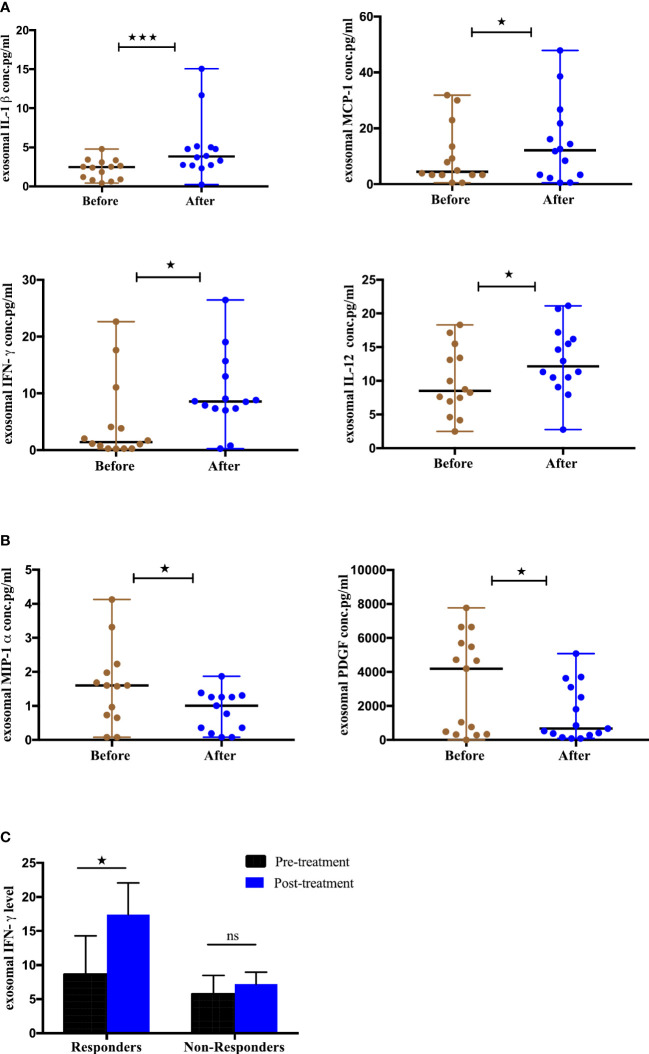
Differential Expression of cytokines from serum-derived exosomes pre- and post-ICI therapy and clinical outcome, **(A)** Significantly upregulated exosomal cytokines and **(B)** Significantly downregulated exosomal cytokines from NSCLC patients before and after anti-PD-1/PD-L1 checkpoint blockade immunotherapy. **(C)** Association of change in exosomal cytokines in responding and non-responding patients (responders, n=7; non-responders=8. Wilcoxon matched-pairs signed-rank test (*p<0.05, **p<0.01, ***p<0.001).

To evaluate the utility of cytokines and chemokines from exosomes as biomarkers to monitor ICIs therapy, we profiled a panel of 44 cytokines/chemokines from the serum-derived exosomes of 15 NSCLC patients at baseline and 4-6 months post-treatment using multiplex bead assay. The mean concentrations are given in (Supplement 3). Our results showed that 6 cytokines were differentially expressed post-treatment (**Figures 9A, B**). Interestingly, IL-1-beta (p=0.0004), MCP-1 (p=0.0342), IFN-γ (p=0.0327) and IL-12 (p=0.0203) were significantly upregulated (**Figure 9A**) while MIP-1α (p=0.0398) and PDGF (p=0.0479) were observably downregulated after ICIs therapy (**Figure 9B**). Thus, our results found there is a change in the expression of cytokines after ICIs therapy and this change may be associated with treatment response. The remaining tested cytokines didn’t show significant differences pre- and post-ICIs therapy. To further assess the association between changes in the expression of these exosomal cytokines and tumor response, the patients were stratified into responding and non-responding groups based on the clinical data of tumor response detected on PET-CT 4-6 months after the initiation of treatment. It was observed that exoIFN-γ (p=0.0156) was significantly upregulated post-treatment in responding patients (**Figure 9C**). Thus, our results showed that IFN-γ from exosomes has the potential to monitor the treatment response of NSCLC patients undergoing ICIs therapy.

## Discussion

Immunotherapy has brought a paradigm shift in cancer treatment, demonstrating significant efficacy and long-term clinical benefits in different cancers, including NSCLC. High tissue PD-L1 expression is currently the golden standard in predicting and selecting patients eligible for immunotherapy. However, the response rate of immunotherapy has been found to be only approximately 20% to 30% in NSCLC (38), comprising only a small subset of NSCLC patients. Therefore, the need to validate effective biomarkers that can facilitate the selection of patients and the monitoring of treatment response for maximum therapeutic effect is critical. Several studies have documented that soluble immune checkpoints and natural killer markers including PD-L1, PD-1, CTLA-4, TIM-3, IDO, NKG2DL, CD27, CD28 etc. can serve as effective biomarkers for monitoring and predicting the treatment response in different cancers as reviewed by Raza et al. (39). However, the biomarker potential of exosomal forms of these markers has not been yet explored in NSCLC. Exosomes have emerged as a promising alternative for cancer detection and treatment monitoring (40, 41) due to their ready availability in most body fluids, advantage of non-invasive/dynamic sampling and high sensitivity and specificity compared to other liquid biopsy forms (22) and the cholesterol-rich lipid bilayer membrane that protects their cargo/content from degradation (24, 25). Since cancer cells actively release exosomes with cancer-promoting content (TEX-related proteins) to mediate intercellular communication within the tumor microenvironment (TME) and promote metastasis and tumor progression (31, 42) these can serve as potential biomarkers for the prediction of treatment response. In this study, we aimed to investigate the utility of T cells, NK immune checkpoint proteins and inflammatory cytokines from serum-derived exosomes in ICIs-treated NSCLC patients to understand the role of these biomarkers in treatment dynamics. We successfully enriched exosomes from the serum of NSCLC patients and verified them according to size, morphology, and expression of exosome specific markers, including CD63, CD81 and HSP70. Moreover, expression of exosomal CD91, reported as a specific marker for lung cancer (43), was also observed in patient samples from this study. In our previous study, we have shown overexpression of CD91 in tissues, cells and exosomes of NSCLC patients (37). Similarly, other published studies have also identified exosomal CD91 as a lung adenocarcinoma specific antigen with high expression reported in stage III/IV lung cancer patients’ sera (43–45). These findings from previous reports thus validate our result indicating the role of CD91 as an NSCLC specific exosomal marker. The main function of CD91, includes its role as a signaling receptor regulating cytokine secretion, phagocytosis, and migration of cells in the immune system. However, it has been reported that high level of serum CD91-expressing exosomes is secreted from stromal cells surrounding lung cancer cells and may be involved in the construction of the tumor microenvironments. However, further larger studies on exosomal CD91 in immunotherapy treated NSCLC patients may shed light on its utility as a diagnostic marker and therapeutic target for NSCLC.

We screened a panel of 13 immune checkpoint markers, 14 NK markers and 44 cytokines and identified that exosomal forms of PD-L1, PD-1, E-Cadherin, MICA, MICB, ULBP1, ULPB3, Siglec7 and IFN-γ could serve as effective biomarkers for anti-PD-1/PD-L1 therapy. PD-L1 is a checkpoint protein, and a type I transmembrane ligand expressed on antigen presenting cells, that binds to its receptor PD-1 on T cells leading to inhibition of effector T-cell activity thereby maintaining immune homeostasis. In cancers, the tumor cells overexpress PD-L1 to suppress the immune response and escape T-cell mediated immune surveillance (46). The tissue expression of PD-L1 and its role as a predictive and prognostic biomarker for immunotherapy is controversial and has several limitations such as invasive sampling, tumor heterogeneity etc (10). Several studies on various cancers such as glioblastoma, melanoma, and head and neck squamous cell carcinoma (HNSCC) have reported on the role of serum-derived exosomal PD-L1 levels as a potential predictive biomarker for ICIs therapies (12, 47, 48). Reportedly, the elevated PD-L1 levels on exosomes were found to be associated with tumor progression in head and neck squamous cell carcinoma (HNSCC) and NSCLC patients (12, 45). In another study involving melanoma and NSCLC patients, the amount of PD-L1 mRNA levels in plasma-derived exosomes was significantly lower in patients with treatment response and significantly higher in patients with disease progression (49). In contrast, high levels of sPD-L1 post-treatment with ICIs were found to be associated with poor response and absence of clinical benefit in NSCLC (50). Our findings confirmed that PD-L1 was found in NSCLC patients’ circulating exosomes and this was consistent with the previous reports.

Additionally, we compared the expression of exoPD-L1 and sPD-L1 from the serum of NSCLC patients and found that exoPD-L1 was expressed at higher levels as compared to free-soluble forms of PD-L1 in serum. A previous study on NSCLC showed similar results, where exoPD-L1was found to be higher as compared to soluble forms and this finding was correlated with advanced tumor stage, larger tumor size, positive lymph node status and distant metastasis (44). Since all the patients in our study had stage 4 tumor with metastasis, this could have been one of the influencing factors for high exoPD-L1. In addition to this, it is well documented that biomolecules released into the serum can be from various sources including immune cells and these biomolecules are prone to enzymatic degradation within the circulation. As compared to soluble serum forms, exosomes carry cancer associated biomolecules in a more stable environment due to their lipid bilayer membrane, as a result of which the carrier biomolecules are sufficiently protected from enzymatic degradation (22, 23). We thus postulated that higher exoPD-L1 observed in our study could be due to these reasons. It also indicates that exoPD-L1 could serve as a potential reliable marker as compared to its soluble-free form.

Further, comparing the exoPD-L1 and tumor PD-L1 IHC profiles, our data showed that exoPD-L1 was present in all 100% of patients while only 71% of patients were tissue PD-L1 positive. This is a powerful finding that indicates that even if tissue-PD-L1 is found to be negative or <1%, exosomal sampling can help to determine the PD-L1 status of patients. Therefore, we postulate that exosomal PD-L1 could be a reliable surrogate marker for tissue-PD-L1. It is well known that tissue heterogeneity plays a significant role in the determination of tissue-PD-L1 status (51) and may, in fact, lead to missed therapeutic opportunities for patients who are tested negative or <1% on tissue. This has been discussed recently in a long-term follow-up study on Checkmate 227, which reported that NSCLC patients with tissue-PD-L1 <1% and treated with nivolumab plus ipilimumab experienced a 5-year overall survival rate of 19% indicating that overall survival outcomes are irrespective of tissue-PD-L1 status (52). This finding allows for further deliberation on the utility of alternative biomarkers such as exosomes that can be utilized as an alternative for tissue-PD-L1 and can reliably determine the PD-L1 status of patients. However, our sample size was small and larger studies can help in a better understanding of this aspect.

Additionally, we measured the baseline levels of PD-L2, another ligand of PD-1, which is expressed in most tumor cells and has an inhibitory effect by interacting with the PD-1 receptor (53). Our results showed higher expression of exoPD-L2 compared to its soluble free form. Further, we also assessed exoPD-L2 levels in tissue PD-L1 positive and negative patients and interestingly found that exoPD-L2 was significantly higher in tissue-PD-L1- patients, supporting a recent parallel study by Sumitomo et al. in which they evaluated the expression of PD-L1 and PD-L2 in NSCLC patients by immunohistochemistry (54). This group found that PD-L1 expression on tumor cells was directly correlated with tumor differentiation while PD-L2 expression was inversely associated, hence suggested that combined assessment of PD-L1 and PD-L2 expression may be considered clinically significant for ICIs-treatment regime in NSCLC patients (54). Thus, our results suggest that NSCLC patients with PD-L1 negative profiles may be evaluated for PD-L2 expression and postulates that PD-L2 may be used as a new target for this group of patients.

Further, we assessed the correlation between exoPD-L1 levels and the outcome of anti-PD-1/PD-L1 therapy. Our findings showed that exoPD-L1 expression was significantly downregulated in responders, while in non-responders, it was significantly upregulated and these findings were consistent with the previous study by Del Re et al. in which they have shown that exoPD-L1 mRNA levels significantly decreased in responding melanoma and NSCLC patients while increased in patients with disease-progression after treatment with anti-PD-1 therapy (49).

The co-inhibitory immune checkpoint PD-1 protein is abundantly expressed in tumor-induced T cells and is triggered by T cell activation (55). We showed that the exoPD-1 expression was significantly higher in the responding patients. Notably, this result was consistent with sPD-1 reported by a previous study in which an increase in sPD-1 concentration after two cycles of nivolumab, was associated with a longer overall survival (56). The most likely explanation for this could be that in responders, the ICIs might prevent the subsequent membrane PD-1/membrane PD-L1 interaction, thereby restoring tumor surveillance by enhancing T cell activity. Further, it is now well recognized, from previous studies, that activated T cells release higher levels of exoPD-1 to block membrane and exoPD-L1-induced inhibition (57).

NKG2DL comprise a group of ligands, including MICA, MICB and ULBP1-6, that bind to NKG2D, a key receptor expressed by natural killer cells and other T cell subsets. Several studies have reported that NKG2D-mediated signaling plays a key role in tumor immunosurveillance and anti-tumor activity (58). Further, studies have reported that tumor exosomes express NKG2D ligands together with other molecules, including TGFβ-1, which trigger the downregulation of the NKG2D receptor expression (59). In contradiction to surface-bound NKG2DL which stimulates anti-tumor activity, the soluble and exosomal forms of these ligands suppress anti-tumor immunity and have been reported to be associated with tumor progression (60). Therefore, we speculated that these exosomal NKG2D ligands should be downregulated in responders post-treatment with ICIs. Our findings were consistent with the soluble forms of such markers reported before. We found significant downregulation of MICA, MICB, ULBP1 and ULBP3 in responders after ICIs therapy. Indeed, our findings support a previous study demonstrating that a reduction in levels of sMICA and sMICB after immunotherapy was associated with improved overall survival in melanoma patients (61).

E-Cadherins are major cell-cell adhesion molecules that play a crucial role in cell growth, invasion, and migration (62). These are primarily synthesized as transmembrane molecules, however, can also be released in membrane cleaved soluble form (sE-cadherin). Tang M et al. reported that in addition to the soluble E-cadherin form, E-cadherin is abundantly released in the exosomes (63). They further demonstrated that exosomal E-cadherin heterodimerizes with VE-cadherin and mediates sequential activation of beta-catenin and NFkB signaling, thereby triggering tumor angiogenesis (63). We measured the levels of exoE-cadherin post-treatment with ICIs and showed that exoE-cadherin levels were significantly downregulated in responders post anti-PD-1/PD-L1 therapy. It has been reported that higher levels of sE-cadherin and exoE-cadherin were correlated with metastasis and tumor progression in several cancers (64, 65). Our findings support also a previous study which showed sE-cadherin was highly expressed in the serum of several cancers, predicting a poor prognosis in these patients (66). Siglec-7 or Sialic acid-binding immunoglobulin-like lectin-7 is an inhibitory receptor expressed on natural killer cells. It belongs to a family of receptors that recognize sialoglycans–sialic acid-containing glycans that are abundantly present on NK cell membranes (67). The siglec-sailoglycon interaction appears to favor tumor immune evasion similar to the PD-1/PD-L1 signaling pathway (67). Reportedly, in metastatic colorectal cancer patients treated with cancer immunotherapy vaccination, a high level of siglec-7 in tumor tissues was linked to shorter overall survival time (68). We have demonstrated that the exosomal levels of siglec-7 were significantly downregulated in responding patients post-ICIs therapy suggesting the use of such molecule as a novel predictive marker to monitor ICIs therapy in NSCLC patients.

Interferon-gamma (IFN-γ) is a pleiotropic cytokine that functions as the principal activator of macrophages and also stimulates natural killer cells and neutrophils. It is critical to both innate and adaptive immunity, and subsequently, promotes antitumor response (69). Several studies have reported that IFN-γ is a valuable candidate for cancer immunotherapy efficacy and thereby proposing its use as a biomarker for monitoring clinical response to ICIs therapy. A prior study reported the higher expression for IFN-γ-related genes in responding patients post ICIs therapy in metastatic melanoma, HNSCC, and gastric cancer (70). Another study has demonstrated that increased IFN-γ levels in the peripheral blood after three months of immunotherapy was associated with better response and overall survival in NSCLC patients (71). Furthermore, many previous studies in several cancers have reported that serum IFN-γ levels were upregulated in responding groups than those in non-responders post-treatment with ICIs therapy (72). Our findings were consistent with these studies as a dramatic increase in exoIFN-γ levels was recorded in responders post-ICIs therapy indicating the role of IFN- γ in disease dynamics and response.

**Figure 10:**
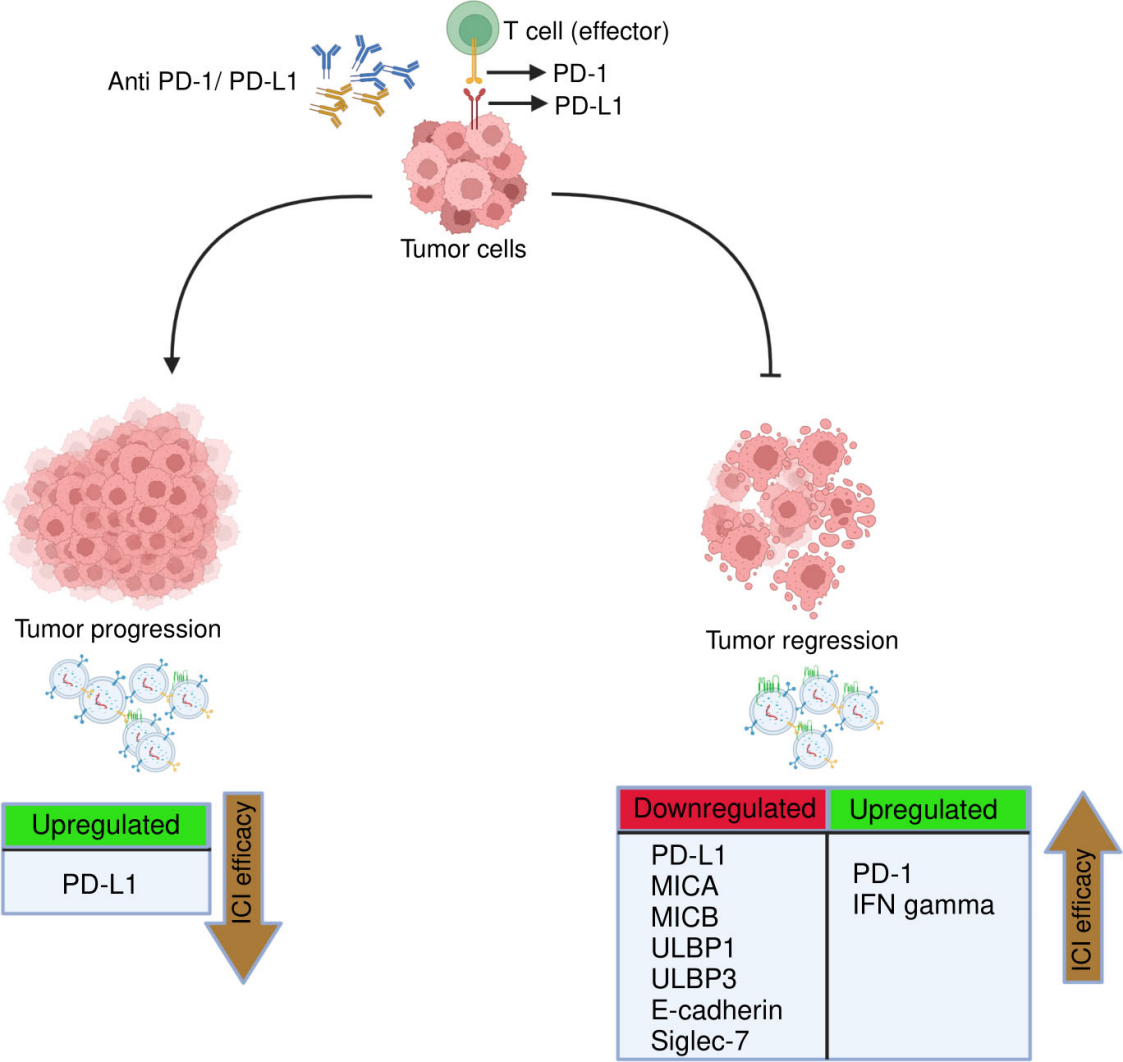
Representation of key immuno-oncological markers and cytokines derived from exosomes associated with clinical response to anti-PD-1/PD-L1 therapy. The efficacy of anti-PD-1/PD-L1 therapy in NSCLC treatment can be monitored/predicted by the presence of following exosomal immune checkpoints, NK markers and cytokines. Tumor regression: Downregulation of exosomal PD-L1, MICA, MICB, ULBP1, ULBP3, E-cadherin, siglec-7 and upregulation of exoPD-1 and exoIFN-γ linked to better ICIs efficacy. Tumor progression: upregulation of exoPD-L1 associated with poor ICIs efficacy.

Our findings indicate that post-anti-PD-1/PD-L1 therapy downregulation of exosomal PD-L1, E-Cadherin, MICA, MICB, ULBP1, ULBP3, Siglec7, and upregulation of exoPD-1, exoIFN-γ were correlated with tumor regression, while upregulation of exoPD-L1 was associated with tumor progression as summarized in **Figure 10**. In addition, we also stratified the patients based on different treatment strategies and evaluated the effect of anti-PD-L1/PD-1 monotherapy and combined therapy on them but we didn’t find any significant difference that could be due to less number of patients in each group. One of the key limitations of this study is the small sample size. Second, although tumor-derived exosomes (TEXs) account for the bulk of serum-derived exosomes, non-tumor cells can also release exosomes into the bloodstream. Our study explored serum-derived exosomes without further isolating the NSCLC sub-population due to the unavailability of a large sample volume, which could have offered more insights into these biomarkers. Additionally, there is a lack of data indicating the relationship of cytokines with either the exosome membrane or the payload; this could be the reason that most of the cytokines were not detected. Thus, further research is needed to study the cytokine profile difference in exosomes isolated by different techniques and between intact and lysed exosomes. Hence, more studies with a larger sample size are needed to validate the clinical potential use of these exosomal biomarkers. Additionally, apart from NSCLC, the possibility of these exosomal markers in monitoring treatment response in ICIs therapies can be explored in other cancers. Also, these tumor-derived exosomal checkpoints possibly may provide great potential treatment targets in combination with additional immunotherapeutic agents in clinical application.

In conclusion, this is the first study to report exosomal immuno-oncological proteins and cytokines as biomarkers to monitor treatment response to ICIs therapy in NSCLC patients. Our findings showed that dramatic downregulation of exosomal PD-L1, E-cadherin, ULBP1, ULBP3, MICA, MICB, Siglec7 and significant upregulation of exosomal PD-1 and IFN-gamma were associated with tumor regression. Additionally, considerable upregulation of exosomal PD-L1 was correlated with tumor progression. In conclusion, this study paves the way towards the potential predictive role of exosomal immune-oncological proteins and cytokines in monitoring treatment response to anti-PD-1/PD-L1 therapy in NSCLC and identifying subsets of patients who are more likely to benefit from these therapies. However, more studies with larger cohorts are warranted to assess the utility of these exosomal biomarkers in clinical practice.

## Data availability statement

The original contributions presented in the study are included in the article/**Supplementary Materials**. Further inquiries can be directed to the corresponding author.

## Ethics statement

The studies involving human participants were reviewed and approved by The Institutional Review Board, Medical Research Center, Hamad Medical Corporation, Doha, Qatar. The patients/participants provided their written informed consent to participate in this study.

## Author contributions

The authors played the following roles in contributing to the manuscript: Conceptualization: SA, AR, SD, RM, AK, AZ, SQ; methodology: SA, AR, SQ, SH, RA-A, IA-B, APa, APh, SV, WM; formal analysis: SA, MI, SS; writing—original draft preparation, SA; writing—review and editing, SA, AR, SD, SQ, MM, UH; visualization: SA, VI; supervision: SD, AR, OK, SQ, SK; project administration: AR, SD; funding acquisition: SD. All authors contributed to the article and approved the submitted version in the manuscript.
