# Supplementary material for: Circulating exosomal immuno-oncological checkpoints and cytokines are potential biomarkers to monitor tumor response to anti-PD-1/PD-L1 therapy in non-small cell lung cancer patients
Source: Front Immunol. 2023 Jan 18;13:1097117. doi: 10.3389/fimmu.2022.1097117 (PMC9890181; doi:10.3389/fimmu.2022.1097117)
Supplement: Supplementary file 1 [file Table_1.docx]

**Supplement 1. Baseline Immuno-oncological protein levels from exosomes and free-soluble forms in serum of NSCLC patients (Mean±SD)**

| **Analytes** | **Serum (pg/ml)** | **Exosome(pg/ml)** | **p-value** |
| --- | --- | --- | --- |
| PD-L1 | 2.256±2.41 | 36.28±12.2 | <0.0001 |
| PD-1 | 53.58±50.49 | 363.93±405.46 | 0.0131 |
| CD152(CTLA4) | 16.32±16.81 | 146.39±203.41 | 0.0008 |
| CD80 | 147.87±246.50 | 338.06±300.35 | 0.0182 |
| IDO | 34.77±24.90 | 123.92±195.58 | 0.0443 |
| Arginase | 49.47±46.69 | 341.53±68.98 | <0.0001 |
| Nectin-2 | 769.79±332.70 | 3174.37±380.41 | <0.0001 |
| NT5E | 300.41±198.08 | 1491.78±1283.50 | <0.0001 |
| Siglec-7 | 326.42±150.76 | 790.37±188.83 | <0.0001 |
| Siglec-9 | 182.78±217.71 | 314.80±330.11 | 0.0335 |
| CD155(PVR) | 2532±1679 | 588.37±507.66 | 0.0004 |
| CD28 | 154.15±152.75 | 569.60±506.60 | 0.0092 |
| GITR | 33.87±69.90 | 329.51±182.77 | <0.0001 |
| MICA | 74.02±53.16 | 733.30±150.74 | <0.0001 |
| CD27 | 1942±3048 | 227.26±176.55 | <0.0001 |
| BTLA | 831.49±2181 | 569.64±605 | 0.1202 |
| LAG3 | 364.70±990 | 1129±2962 | 0.7820 |
| PD-L2 | 3562±9907 | 1571±2781 | 0.7414 |
| E-Cadherin | 50.82±45.45 | 15449±50253 | 0.9999 |
| CD137 | 78.93±103 | 3368±13102 | 0.3060 |
| MICB | 65.21±43.2 | 67.44±11.93 | 0.4212 |
