# Supplementary material for: Circulating exosomal immuno-oncological checkpoints and cytokines are potential biomarkers to monitor tumor response to anti-PD-1/PD-L1 therapy in non-small cell lung cancer patients
Source: Front Immunol. 2023 Jan 18;13:1097117. doi: 10.3389/fimmu.2022.1097117 (PMC9890181; doi:10.3389/fimmu.2022.1097117)
Supplement: Supplementary file 2 [file Table_2.docx]

**Supplement 2. Changes in the exosomal immuno-oncological proteins before and after ICI therapy in NSCLC patients.**

| **Analytes** | **Pre-ICI therapy** | **Post-ICI therapy** | **p-value** |
| --- | --- | --- | --- |
| PD-1 | 363.92±405 | 1748±1580 | 0.0006 |
| PD-L2 | 1572±2781 | 6327±16097 | 0.0038 |
| PD-L1 | 45.247±39.03 | 115.25±233 | 0.0624 |
| BTLA | 569.64±606 | 1018±1162 | 0.0358 |
| IDO | 79.52±84.79 | 124.16±114 | 0.0413 |
| HVEM | 254.46±134 | 644.73±1330 | 0.0353 |
| GITR | 470.68±591 | 1663.71±3400 | 0.0076 |
| Siglec-7 | 761.72±132 | 678.90±120 | 0.0250 |
| CD96 (Tactile) | 1583±586 | 1111±305 | 0.0079 |
| CD73 (NT5E) | 1508±1241 | 781±278 | 0.0004 |
| CD155(PVR) | 560.37±504 | 282.87±207 | 0.0042 |
| MICA | 728.20±147 | 660.37±53 | 0.0457 |
| MICB | 51.44±11.93 | 44.08±7.65 | 0.0029 |
| ULBP-1 | 2322±2424 | 733±1033 | 0.0215 |
| ULBP-3 | 765.43±614 | 393.59±390 | 0.0063 |
| CD137 | 3368±13102 | 1159±3421 | 0.1391 |
| CD152 | 2157±8294 | 515±1310 | 0.2435 |
| CD27 | 237.04±175 | 257.84±148 | 0.7819 |
| CD28 | 569.59±506 | 1584±3090 | 0.1324 |
| CD80 | 11244±44968 | 1297±2805 | 0.1594 |
| LAG3 | 1129±2962 | 1483±2950 | 0.2524 |
| Arginase-1 | 346.39±69.74 | 358.79±81.69 | 0.7119 |
| E-cadherin | 15449±50253 | 3458±4181 | 0.0714 |
| Nectin2(CD112) | 3171±368 | 3085±355 | 0.7467 |
| Siglec9 | 352.29±355 | 313.90±371 | 0.8702 |
