# Supplementary material for: Circulating exosomal immuno-oncological checkpoints and cytokines are potential biomarkers to monitor tumor response to anti-PD-1/PD-L1 therapy in non-small cell lung cancer patients
Source: Front Immunol. 2023 Jan 18;13:1097117. doi: 10.3389/fimmu.2022.1097117 (PMC9890181; doi:10.3389/fimmu.2022.1097117)
Supplement: Supplementary file 3 [file Table_3.docx]

**Supplement 3. Immuno-oncological cytokines before and after ICI therapy**

**(Mean±SD)**

| **Analytes** | **Pre-treatment** | **Post-treatment** | **p-value** |
| --- | --- | --- | --- |
| IL-1 beta | 2.16±1.27 | 4.71±3.97 | 0.0004 |
| MCP-1 | 9.90±10.67 | 14.87±14.50 | 0.0342 |
| IFN-gamma | 4.78±7.16 | 9.98±6.83 | 0.0327 |
| IL-12 | 9.84±4.93 | 12.97±5 | 0.0203 |
| MIP-1-alpha | 1.54±1.14 | 1.12±1.13 | 0.0398 |
| PDGF | 3271±2861 | 1552±1639 | 0.0479 |
| Basic FGF | 80.96±29.20 | 87.30±48.91 | 0.9780 |
| GCSF | 552.61±661 | 568.01±426 | 0.9780 |
| HGF | 267.37±160 | 244.30±131 | 0.7615 |
| IFN-Alpha2 | 23.19±23.60 | 26.69±22.68 | 0.7148 |
| IL-1 Alpha | 33.95±26.45 | 76.82±114 | 0.1937 |
| IL-1-RA | 422.99±230 | 596.50±653 | 0.4887 |
| IL-2 | 18.37±11.24 | 27.10±25.57 | 0.1876 |
| IL-2 RALPHA | 66.67±75.68 | 90.07±108 | 0.8552 |
| IL-4 | 1.68±1.85 | 1.68±1.45 | 0.9861 |
| IL-6 | 9.35±5.29 | 9.93±7.34 | 0.7615 |
| IL-7 | 15.77±14.95 | 20.88±15.27 | 0.1008 |
| IL-8 | 6.25±6.37 | 8.40±9.52 | 0.4212 |
| IL-9 | 750.61±518 | 659.91±400 | 0.4543 |
| IL-10 | 8.30±3.75 | 8.66±4.42 | 0.5995 |
| 1L-12(P40) | 353.56±185 | 360.43±193 | 0.8904 |
| IL-16 | 42.88±40.70 | 50.81±67.84 | 0.8040 |
| IP-10 | 184.79±163.71 | 218.12±145 | 0.5245 |
| LIF | 86.74±129 | 126.03±253 | 0.8904 |
| MCP-3 | 13.20±12.30 | 16.91±16.16 | 0.1677 |
| M-CSP | 27.02±8.75 | 29.17±10.85 | 0.9780 |
| MIF | 850.45±905 | 864.80±729 | 0.9341 |
| MIG | 154.43±124.66 | 181.60±150 | 0.8904 |
| MIP-1 Beta | 464.01±317 | 406.97±244 | 0.4887 |
| Beta NGF | 15.55±12.29 | 19.12±21.92 | 0.5417 |
| RANTES | 14148±18918 | 6633±8813 | 0.2769 |
| SCF | 19.08±9.65 | 24.67±29.55 | 0.9515 |
| SCGF-Beta | 61334±41542 | 55471±38303 | 0.5614 |
| SDF-1 Alpha | 1429±1321 | 1060±896 | 0.2524 |
| TNF Alpha | 30.50±16.25 | 38.15±26.21 | 0.4887 |
| TNF Beta | 586.23±413 | 519.86±346 | 0.5995 |
| TRAIL | 13.10±8.88 | 17.30±9.36 | 0.1514 |
| VEGF | 926.49±734 | 881.27±930 | 0.5879 |
